# Extracorporeal Photopheresis in Pediatric and Adult Patients with Graft-Versus-Host Disease

**DOI:** 10.3390/jcm13175192

**Published:** 2024-09-01

**Authors:** Alexandra Ionete, Marius Surleac, Mihaela Uta, Zsofia Varady, Ana Maria Bica, Cristina Georgiana Jercan, Anca Colita, Daniel Coriu

**Affiliations:** 1 Stem Cell Bank, Fundeni Clinical Institute, 022328 Bucharest, Romania; 2 Faculty of General Medicine, University of Medicine and Pharmacy “Carol Davila”, 020021 Bucharest, Romania; 3 Department of Molecular Genetics, National Institute for Infectious Diseases “Matei Bals”, 021105 Bucharest, Romania; 4 Department of Hematology, Fundeni Clinical Institute, 022328 Bucharest, Romania; 5 Department of Hematology-Bone Marrow Transplant Unit, Fundeni Clinical Institute, 022328 Bucharest, Romania; 6 Pediatrics Clinic, Fundeni Clinical Institute, 022328 Bucharest, Romania

**Keywords:** GVHD, ECP, methylprednisolone, ruxolitinib, allogeneic hematopoietic stem cell transplantation

## Abstract

**Background/Objectives**: Graft-versus-host disease (GVHD) is a severe complication of allogeneic hematopoietic stem cell transplantation (allo-HSCT) resulting from histocompatibility differences between donor and host cells leading to inflammation, tissue damage, and compromised patient outcome. Extracorporeal photopheresis (ECP) is considered as a second-line treatment administered to patients with GVHD who do not respond to corticosteroid treatment or who experience a relapse after an initial response and are therefore classified as steroid refractory (SR). The aim of this study is to evaluate the clinical response rates in both pediatric and adult patients with acute (a) or chronic (c) GVHD and to assess the effectiveness of ECP using the real-world data from a single center. **Methods**: We performed a retrospective study on 30 patients, including 11 pediatric and 19 adult patients who were treated with ECP as a second-, third-, or fourth-line therapy for (a) and (c) GVHD, alongside corticosteroids and other immunomodulatory medications. The median time from aGVHD onset to ECP was 11.5 days (range: 3 days–9 months), while for cGVHD, the median time was 90 days (range: 2 days–9 months). **Results**: The overall response rate (ORR) in the aGVHD patient population was 60% with a median of 9 procedures (range: 2–20). For cGVHD patients, the ORR was 70% after a median of 23.5 ECP procedures (range: 8–43). Most patients had skin involvement, with ECP achieving an ORR of 81.8% in aGVHD and 77.7% in cGVHD cases. **Conclusions**: ECP is a beneficial therapy for patients with (a) and (c) GVHD who have not responded to corticosteroids and other forms of immunosuppressive therapy. Specifically, ECP demonstrated efficacy in improving skin and oral symptoms and permitted reductions in or the elimination of their corticosteroid usage. The study found that extending the duration of ECP treatment was associated with better outcomes, and no detectable complications were observed over a 38-week period.

## 1. Introduction

Graft-versus-host disease (GVHD) is a serious immune system complication that can occur after allogeneic hematopoietic stem cell transplant (allo-HSCT), leading to significant problems with disability and reduced survival. Corticosteroids are the primary treatment option for GVHD. However, their effectiveness is limited, and many patients require second- and third-line, as well as sometimes fourth-line therapies. These may include immunosuppressants, such as mycophenolate mofetil and sirolimus; extracorporeal photopheresis (ECP); anti-TNF-α and anti-IL-2R antibodies; selective Janus kinase (JAK) 1 and 2 (ruxolitinib) inhibitors; and cell therapies, including mesenchymal stem cells [1,2].

ECP gained FDA approval in 1988 as the first cancer immunotherapy. ECP has a wide range of clinical applications, including cutaneous T-cell lymphoma CTCL (notably mycosis fungoides and Sézary syndrome), acute (a) and chronic (c) GVHD, organ and tissue transplantation (e.g., cardiac and lung allograft rejection), systemic sclerosis, systemic lupus erythematosus, rheumatoid arthritis, Crohn’s disease, and pemphigus vulgaris [3,4,5,6,7,8,9,10,11,12,13].

Although the precise mechanism of ECP remains unclear, it is hypothesized that following UVA exposure, activated 8-MOP interacts with DNA strands within mononuclear cells, inhibiting DNA replication and inducing cellular apoptosis [14,15,16]. Both 8-MOP and UVA light react with various cellular macromolecules, including cell surface molecules and cytoplasmic components [17]. ECP induces apoptosis in lymphomononuclear cells, which are subsequently phagocytized by macrophages, and promotes monocyte differentiation into immature dendritic cells possibly due to transient adhesion of monocytes to the plastic tubing of the photoinactivation kit [18,19,20]. Despite only treating a small percentage of circulating T lymphocytes, ECP effectively induces immune tolerance without generalized immunosuppression [21]. The possible mechanisms by which ECP induces immune tolerance include inhibition of proinflammatory cytokines, increased secretion of anti-inflammatory cytokines, decreased stimulation of effector T cell, enhanced effector cell apoptosis, and increased generation of regulatory T cells [22]. Apoptotic cells influence dendritic cell (DC) maturation, with early apoptotic cells leading to semi-mature DCs that promote tolerance. Studies indicate that DCs in ECP-treated cGVHD patients express immature characteristics, and ECP increases regulatory T cell populations, which suppress effector T cell activity and reduce inflammation, thereby improving patient outcomes [23].

The objective of the study was to assess the clinical response rates and safety profile of ECP treatment in both pediatric and adult patients with aGVHD and cGVHD who did not respond to corticosteroids and other immunosuppressive treatments and to evaluate its potential benefits when used as a second-line or later therapy.

## 2. Materials and Methods

### 2.1. Patient Population and Inclusion Criteria

From May 2015 to December 2023, a total of 30 patients, including both adults (*n* = 19) and pediatric individuals (*n* = 11), were treated with ECP as a second-, third-, or fourth-line therapy for GVHD at Fundeni Clinical Institute. It is noteworthy that only the Fundeni Clinical Institute provides ECP treatment among all the centers in Romania that perform allo-HSCT. The reported investigations were performed according to the principles of the Declaration of Helsinki.

Inclusion criteria for aGVHD: Patients must have a confirmed diagnosis based on clinical and histopathological criteria, with the disease graded as II to IV. Patients should be refractory to corticosteroids or have contraindications for standard first-line treatments, such as high-dose corticosteroids, and must show an inadequate response to initial steroid therapy, defined as progression or no improvement in GVHD after 3–5 days. Patients must be hemodynamically stable and capable of tolerating the ECP procedure.

Inclusion criteria for cGVHD: Patients must have a confirmed diagnosis, which is usually determined by clinical symptoms and physical examination. The condition must be moderate to severe and either resistant to or reliant on corticosteroid treatment, affecting the skin, liver, oral mucosa, and lungs. ECP is considered for those who have not responded to or cannot taper off corticosteroids and other immunosuppressive therapies, particularly if they need to reduce long-term immunosuppressive use due to side effects or complications. Patients should also have a significantly impaired quality of life despite standard treatments and must be stable enough to undergo ECP with proper venous access.

General considerations for ECP include no strict age limits, but patients should have a performance status that permits the procedure. Acceptable coagulation parameters are necessary to avoid complications, and informed consent must be obtained from patients or their guardians after they have been thoroughly informed about the risks and benefits of ECP.

### 2.2. GVHD Staging and Response Evaluation

aGVHD was evaluated using staging criteria from the EBMT Handbook, which considers the severity of symptoms in four target organs: skin, liver, upper GI, and lower GI. Skin involvement is assessed by the extent of maculopapular rash coverage (ranging from no rash to generalized erythroderma with bullous formation). Liver involvement is measured using bilirubin levels (ranging from less than 2 mg/dL to greater than 15 mg/dL). Upper GI symptoms include nausea, vomiting, and anorexia, while lower GI symptoms are assessed based on stool output, varying from less than 500 mL/day to severe conditions like grossly bloody stool. These stages (0 to 4) are combined to determine the overall grade of aGVHD, which ranges from Grade 0 (no symptoms) to Grade IV (severe involvement of skin, liver, or lower G)I [24].

cGVHD severity was determined by the number and severity of organs affected. The overall severity is classified into mild, moderate, or severe categories. Mild cGVHD involves 1–2 organs without lung involvement, moderate cGVHD involves more than 3 organs or mild to moderate lung involvement, and severe cGVHD is characterized by severe involvement of at least three organs, including moderate or severe lung disease [24].

A complete response (CR) was defined as the total resolution of all clinical indicators of GVHD. A partial/favorable response (PR) was characterized by at least a 50% improvement in clinical indicators of GVHD without achieving a CR and the ability to reduce steroid use by 50% without recurrence of symptoms or clinical indicators. Non-responding (NR) status was defined as no improvement in symptoms and/or clinical indicators or early death due to GVHD.

### 2.3. ECP Treatment

In this study, the “off-line” technique was used, which involves two distinct steps [25]: (i) Collection of mononuclear cells (MNCs, buffy-coat) using continuous flow cell separators. This procedure was initially performed using the Cobe Spectra (Terumo BCT) until 2017. Then, the Optia Spectra (Terumo BCT) was used under the CMNC protocol. MNC apheresis was performed using a central venous catheter (CVC) with anticoagulant citrate dextrose solution A (ACD-A) as the anticoagulant, at a ratio of 1:12 to 1:13, treating a total blood volume (TBV) between 1.5 to 2 times the patient’s TBV. For children weighing less than 25 kg, a Custom Prime with 300 mL irradiated erythrocyte mass was used for apheresis. (ii) Ex vivo treatment. The apheresis-harvested buffy-coat was incubated ex-vivo with 8-MOP (at a final concentration of 200 ng/mL) and then exposed to UVA irradiation (2 J/cm^2^). This step was initially performed using the MTS UVA Pit device (PIT Medical Systems GmbH) until February 2022 and subsequently performed using the Lumi-Light (Pelham) device starting on January 2023. The final product was reinfused into the patient on the same day. The initiation and frequency of ECP sessions varied widely, with a median of 11 sessions and a range of 2 to 43 sessions.

In patients with stage 3 or 4 aGVHD and moderate or severe cGVHD, ECP started with three sessions/week. The procedure was performed for one month or according to the evolution of the GVHD. Then, the frequency of sessions decreased to two per week, one per week, one per three weeks, followed by one per month, with each frequency maintained for a certain number of weeks, depending on the evolution of the disease. For other forms of GVHD, ECP started with two sessions per week. In parallel with the decrease in the frequency of ECP sessions, the dose of immunosuppressive medication also decreases. Eleven patients were treated with second-line ECP therapy (nine with aGVHD, two with cGVHD), twelve with third-line therapy (eight with aGVHD, four with cGVHD), and seven with fourth-line therapy (three with aGVHD, four with cGVHD).

### 2.4. Statistical Methods

The baseline characteristics were reported using descriptive statistics. Chi-square or Fisher’s exact tests were used to analyze factors associated with the overall response (OR). Statistical significance was considered for *p*-values < 0.05. All analyses were done using GraphPad Prism for Windows (v.9) software (GraphPad Software, San Diego, CA, USA).

## 3. Results

### 3.1. Patient Characteristics 

A retrospective medical evaluation was performed utilizing anonymized patient-level data extracted from the electronic medical records of 30 individuals who received ECP treatment for acute (*n* = 20) or chronic GVHD (*n* = 9) and one individual with overlap syndrome at the Department of Bone Marrow Transplantation Unit and Stem Cell Bank (Table 1). The overlap case was included in the analysis as chronic GVHD because the patient presented cGVHD characteristics. At the initiation point of ECP treatment, all patients had previously received systemic corticosteroids and other immunosuppressive therapies for GVHD. Out of the total, 29 patients received peripheral blood stem cell grafts for conditions such as acute myeloid leukemia (AML), acute lymphoblastic leukemia (ALL), aplastic anemia (AA), Hodgkin lymphoma (HL), non-Hodgkin lymphoma (NHL), multiple myeloma (MM) and myelodysplastic syndrome (MDS), except for one patient who received a bone marrow graft.

One patient received a bone marrow graft from a mismatched unrelated donor (9/10). On day +22, the patient developed acute GVHD with skin involvement. ECP sessions were initiated 10 days after the onset of GVHD, leading to complete resolution of the acute skin GVHD. However, the patient later passed away from other causes. The other patients received either a matched unrelated donor (MUD, 50%) or a matched sibling donor (MSD, 26.67%), and 23.33% had a haploidentical donor (HAPLO). The median time from aGVHD onset to ECP was 11.5 days (range: 3 days–9 months), while for cGVHD, the median time was 90 days (range: 2 days–9 months). The median age of the pediatric patient cohort was 8 years, with a gender distribution of 45.45% females (5 patients) and 54.55% males (6 patients). In contrast, the adult patient cohort had a median age of 37 years, with a significant gender imbalance of 68.42% females (13 patients) versus 31.58% males (6 patients). In our study, the cell collection efficiency yielded an average of 38.1 × 10^9^/L white blood cell (WBCs) using the “offline” system. The total average procedure time was 159 min [126.7–199.3], with an average buffy coat volume of 125 mL [57–222].

aGVHD was graded II for two patients (10%), III for six (30%), and IV for twelve patients (60%). The distribution of cGVHD cases is relatively balanced across the staging categories. The moderate stage has the highest occurrence at 40%. Both mild and severe stages each represent 30% of the total cases (Table 2).

### 3.2. Adverse Events after ECP

WBC counts decreased during the ECP treatment, dropping from a median of 4.4 × 10^9^/L (range: 1.7–9 × 10^9^/L) before treatment to 3.95 × 10^9^ g/dL (range: 1.1–8.8 × 10^9^/L) after treatment (*p* = 0.0251). Hemoglobin levels also decreased from a median of 9 g/dL (range: 7.6–15.2) before treatment to a median of 8.6 (range: 6.8–16.4) after treatment (not statistically significant, ns). Hematocrit levels remained stable, with a median of 26.1% (range: 21.1–43.6) before treatment, dropping to 24.2% (range: 19.6–39.6) after treatment (*p* = <0.0001). Thrombocyte counts decreased from a median of 69.65 × 10^9^/L (range: 12–409.6) before treatment to a median of 52.7 × 10^9^/L (range: 10–327.5 × 10^9^/L) after treatment (*p* = <0.0001). Previous research has revealed comparable findings on blood parameters [26]. While ECP can have an impact on the levels of hemoglobin, hematocrit, leukocytes, lymphocytes, and thrombocytes, not all changes are clinically significant or solely attributable to ECP. However, it is important to note that there is a substantial risk of anemia (maximum grade 3 using CTCAE grading) and thrombocytopenia emerging over time in patients receiving ECP treatment, emphasizing the need for consistent monitoring of blood parameters. Despite these occurrences, the adverse events were managed effectively, and no grade 4 or higher hematologic toxicities were reported.

One non-hematological adverse effect observed during extracorporeal photopheresis (ECP) was hypocalcemia, which was classified as grade 2 according to CTCAE. For moderate to severe hypocalcemia, intravenous calcium gluconate was commonly administered

### 3.3. Clinical Response of aGVHD after ECP Treatment 

ECP treatment for aGVHD showed an overall response rate (ORR) of 60%, including either a complete (CR) or partial response (PR), and an overall survival rate (OS) of approximately 40% (with three patients dying from causes unrelated to ECP). Notably, the use of ECP as a second-line therapy was associated with better responses in aGVHD patients (Table 3).

The majority of patients had skin involvement (66.7%). Regarding aGVHD, 20% of patients had grade I-II (*n* = 4), grade III 15% (*n* = 3), or grade IV 20% (*n* = 4) involvement, with an ORR of 81.8%. All patients with grades I-III of aGVHD achieved a CR. Among those with grade IV aGVHD, two patients obtained an ORR. This suggests that while ECP can still be beneficial for severe cases, it may not be as effective as it is for less severe cases. The six-month OS for patients with skin aGVHD was 72%, with the one-year survival rate dropping to 40%. It is important to note that three patients passed away due to causes unrelated to GVHD had responded to ECP therapy. Next in frequency were cases of aGVHD with 33.3% gastrointestinal involvement (GI). Of these, 15% of patients had grade III involvement (*n* = 3) with two achieving a CR while 30% had grade IV involvement (*n* = 6). At these higher severity levels, ECP’s effectiveness was lower, with only one patient achieving a partial response (PR).

### 3.4. Clinical Response of cGVHD after ECP Treatment

For cGVHD, 70% of patients had an ORR to treatment, with a higher OS of 60% (with one patient dying from other causes). In the case of skin cGVHD, 20% of patients had mild (*n* = 2), moderate 40% (*n* = 4), or severe 30% (*n* = 3) involvement. Additionally, among cGVHD patients with skin involvement, 13.3% experienced ocular involvement, 13.3% mucous membrane, 13.3% gastrointestinal, 6.7% connective tissue, and 3.3% genital involvement. In total, 50% (*n* = 5) of patients with moderate and severe skin cGVHD achieved a CR with an ORR of 77.7%. The OS at six months was 100%, while the one-year survival rate was 78%, with one patient who responded to ECP treatment passing away due to causes unrelated to GVHD. Only one cGVHD patient initially presented with moderate lung involvement (3.3%), while two others developed pulmonary GVHD during the course of their disease. All these patients did not survive.

Among all patients with GI involvement, 50% had a *Clostridium difficile* infection (which constitutes 16.7% of the total patient cohort). All patients with this infection did not survive. Eleven patients were treated with second-line ECP therapy (nine with aGVHD, two with cGVHD), eleven with third-line therapy (seven with aGVHD, four with cGVHD), and eight with fourth-line therapy (four with aGVHD, four with cGVHD) (Table 3).

Patients with aGVHD or cGVHD who started ECP treatment within two weeks of GVHD onset responded in 50% of cases. In contrast, patients who began ECP treatment later than two weeks achieved a 75% ORR (Table 4). For those who started ECP treatment earlier, the transition from a femoral central venous catheter (CVC) to a “long life” CVC typically resulted in a longer pause in ECP sessions. This interruption could be a possible explanation for the lower response rates observed in patients who initiated treatment within two weeks of GVHD onset. Patients who achieved a CR to ECP treatment began therapy an average of 19.11 days after GVHD onset. Those with a PR started treatment an average of 92.89 days after GVHD onset, which is significantly later compared to the complete responders. Patients who did not respond to ECP started treatment an average of 44.67 days after GVHD onset. Despite these differences, statistical analysis using the Bonferroni test revealed no significant variations in response based on the timing of ECP initiation (*p* > 0.05 for all comparisons). These findings suggest that the timing of ECP initiation relative to GVHD onset does not significantly impact the treatment response. However, larger studies are needed to confirm these results and to investigate other factors that might affect ECP treatment outcomes.

The clinical responses following ECP were not correlated with the baseline GVHD or with the age patients in the pediatric and adult groups. Furthermore, there was no correlation observed between clinical responses and sex and HLA match.

On May 24, 2019, the FDA approved ruxolitinib for SR-aGVHD; in September 2021, it was approved for SR-cGVHD in adult and pediatric patients 12 years and older [27,28]. In our cohort, 23% (*n* = 8) of patients were treated with ruxolitinib as a third- or fourth-line therapy after initiation of ECP treatment. For seven patients, ECP and ruxolitinib were administered simultaneously. In total, 50% of these patients (*n* = 4) achieved an ORR to the treatment. For one patient, ruxolitinib replaced ECP. He developed conjunctival and cutaneous GVHD and initially responded completely after 43 sessions of ECP. However, the skin response was not maintained after discontinuing ECP. The cutaneous GVHD completely remitted after the introduction of ruxolitinib.

## 4. Discussion

ECP therapy emerges as a promising second-line treatment that is significantly associated with better responses. These insights can inform treatment decisions and strategies for managing GVHD, emphasizing the importance of considering ECP therapy in second-line treatment plans. Considering the favorable safety profile of ECP and its minimal drug interactions, it would be beneficial to modify the treatment protocol to incorporate methylprednisolone (as a primary first-line therapy) in conjunction with ECP from the outset (rather than as a second or third line of treatment, as we currently employ) to enhance complete response rates.

ECP can be performed using two different techniques: the closed system (“on-line system”, e.g., Therakos, Inc.) and the open system (“off-line system”). Although the closed system (in-line) offers several advantages (no risk of contamination of the administered product, shorter procedure time, 1.5–2 h), it has a considerable disadvantage when it comes to children as it requires children to weigh at least 40 kg [29].

ECP has demonstrated high efficacy for patients with skin involvement in aGVHD. Specifically, all patients with grade I–III aGVHD achieved complete remission, indicating that ECP is particularly effective for treating skin manifestations at these severity levels. However, for patients with grade IV skin aGVHD, the response was less consistent. This shows that while ECP remains beneficial for severe cases, it is not as universally effective as for less severe grades. The outcomes observed in our study for both refractory (a) and (c) skin GVHD treated with ECP are consistent with those reported in the existing literature. Numerous studies involving varying patient cohorts were discussed in a literature review, reporting response rates for refractory aGVHD to ECP as a second-line therapy ranging from 60% to 100%. Similarly, studies on refractory cGVHD have documented response rates to ECP as a second-line therapy ranging from 31% to 100% [30]. In our study, we observed an ORR of 81.8% for aGVHD and 77.7% for cGVHD skin involvement.

In cases of gastrointestinal involvement, ECP treatment was more effective for grade III aGVHD, though with a lower response rate compared to skin involvement. For grade IV GI aGVHD, ECP was less effective, compounded by complications, such as *Clostridium difficile* infection. This highlights a critical complication that negatively impacts the efficacy of ECP, as the presence of *Clostridium difficile* infection severely affects both survival and response to treatment.

Lung involvement did not respond to ECP therapy, which is consistent with the literature reports [31]. In our group, three patients had pulmonary GVHD, none of whom survived. This included one patient with isolated pulmonary GVHD and two others who developed pulmonary GVHD during the course of their disease, initially starting ECP for skin involvement. One patient died from a relapse of the underlying leukemia rather than from GVHD, highlighting that GVHD is not always accompanied by the graft-versus-leukemia (GVL) effect. [32].

Although the peripheral venous access is preferred for apheresis, all of our patients required central venous catheter in order to perform apheresis procedures for ECP. Often, for many patients, a central venous catheter was placed in the femoral vein at the beginning of the apheresis sessions. During the apheresis, we faced two types of problems: catheter-related problems (insufficient flow rate up to blockage in some cases) and citrate-related problems (hypocalcemia, solved by increasing the flow rate of prophylactic calcium gluconate infusion). Another issue linked to central venous access is the elevated risk of bacteremia due to potential transmission of skin microbiota into the bloodstream, thereby increasing the risk of *Clostridium difficile* infection, especially in patients with gastrointestinal involvement, which was correlated with high mortality in our cohort. *Clostridium difficile* infections are a frequent complication in these patients due to the numerous antibiotic regimens they receive during the course of their disease. Managing *Clostridium difficile* infections in daily practice proved to be quite challenging. During apheresis, the back-up device was transported to the patient’s ward and remained there until the ECP sessions concluded. This procedure required both a doctor and a nurse to perform the treatment in the isolation room, which was a logistical challenge for our small team. Simultaneously, medical personnel needed to be present in the dedicated room to manage other apheresis procedures. Therefore, it is essential to investigate whether *Clostridium difficile* is the trigger for the intestinal inflammation that subsequently activates lymphocytes. To address microbiota imbalances and treat GVHD triggered by this inflammation, we are exploring the use of fecal transplantation for these patients.

Our study can be considered a “real-life” study because it reflects the practical challenges encountered in clinical settings. Despite our intention to use ECP as the second line of therapy, technical constraints often prevented this from occurring as planned. Specifically, initiating ECP sessions requires the placement of a central venous catheter (CVC). In cases where the patient’s clinical condition precluded timely CVC placement, patients often had to proceed with second- or even third-line therapies instead. This realistic approach highlights the complexities and limitations of implementing ECP in routine clinical practice.

## 5. Conclusions

Despite limitations, such as heterogeneity, small numbers in the patient cohort, and the concomitant use of other immunosuppressive medications or ruxolitinib, ECP demonstrated better response rates with extended treatment durations and no detectable complications over a 38-week period. As for skin aGVHD, progress is seen from one session to the next. In addition, this type of GVHD determines the most favorable response to ECP therapy. However, the absence of standardization in the application of ECP, including methods such as “online” vs. “offline” systems, initiation timing, treatment schedules, and dose reduction protocols, remain a significant obstacle. Despite these uncertainties, ECP’s favorable safety profile makes it a promising alternative to other immunosuppressive therapies. Consequently, clinical trials are essential to determine the optimal use of ECP in allo-HSCT, including its prophylactic use in haploidentical transplants and its use as part of initial combination therapy. Further well-designed studies are necessary to better understand the mechanism of ECP, optimize treatment timing, and identify biomarkers to determine patient benefit.

## Figures and Tables

**Table 1:** Patient characteristics.

Patient Characteristics	No. (*n* = 30)	%
Age, years		
Median [range]	23.5 years [2–60]	
Patient sex		
Female	18	60.00
Male	12	40.00
Primary disease		
ALL	11	36.67
AML	8	26.67
MDS	4	13.33
AA	3	10.00
HL	2	6.67
NHL	1	3.33
MM	1	3.33
HLA typing		
Matched unrelated (MUD)	15	50.00
Matched sibling donor (MSD)	8	26.67
Haploidentical donor (HAPLO)	7	23.33
Graft source		
Peripheral blood	29	96.67
Bone marrow	1	3.33
Days from aGVHD onset to ECP		
Median [range]	11.5 days [3 days–9 months]	
Days from cGVHD onset to ECP		
Median [Range]	90 days [2 days–9 months]	
Treatment prior to ECP initiation		
Corticosteroids	11	36.67
Corticosteroids and CNIs	12	40
Corticosteroids + CNIs + MMF/Rap	7	23.33

ALL, acute lymphoblastic leukemia; AML, acute myeloid leukemia; MDS, myelodysplastic syndrome; AA, aplastic anemia; HL, Hodgkin lymphoma; NHL, non-Hodgkin lymphoma; MM, multiple myeloma, CNIs, calcineurin inhibitors; MMF, mycophenolate mofetil; Rap, rapamycin.

**Table 2:** Staging of aGVHD and cGVHD.

**aGVHD Staging**	**I-II**	**III**	**IV**
	2 (10%)	6 (30%)	12 (60%)
cGVHD staging	Mild	Moderate	Severe
	3 (30%)	4 (40%)	3 (30%)

**Table 3:** ECP line treatment response.

**aGVHD Response**	**2th Line**	**3th Line**	**4th Line**
CR/PR	7	4	1
NR	2	3	3
cGVHD Response	2th Line	3th Line	4th Line
CR/PR	1	4	2
NR	1	0	2

CR, complete response; NR, no response; PR, partial response.

**Table 4:** Time from ECP initiation to response.

	Response to ECP			GVHD Type		
Time (weeks)	CR	PR	NR	Acute	Chronic	Overlap
0	0	0	0	0	0	0
0.5	2	0	1	2	0	1
1	2	1	4	6	1	0
1.5	3	1	1	5	0	0
2	1	0	2	3	0	0
3	3	0	0	2	1	0
6	0	1	0	0	1	0
8	1	1	1	2	1	0
16	0	1	0	0	1	0
20	0	1	0	0	1	0
24	0	1	0	0	1	0
38	0	1	1	0	2	0

## Data Availability

The data that support the conclusions of this research are accessible from the corresponding author upon reasonable request and taking into account privacy concerns.
